# A (H1N1) pdm09 HA D222 variants associated with severity and mortality in patients during a second wave in Mexico

**DOI:** 10.1186/1743-422X-10-41

**Published:** 2013-01-31

**Authors:** Joel A Vazquez-Perez, Pavel Isa, Darwyn Kobasa, Christopher E Ormsby, Jose E Ramírez-Gonzalez, Damaris P Romero-Rodríguez, Charlene Ranadheera, Yan Li, Nathalie Bastien, Carissa Embury-Hyatt, Elizabeth González-Duran, Gisela Barrera-Badillo, Yuria Ablanedo-Terrazas, Edgar E Sevilla-Reyes, Marina Escalera-Zamudio, Ana G Cobián-Güemes, Irma Lopez, Joanna Ortiz-Alcántara, Celia Alpuche-Aranda, Jose R Perez-Padilla, Gustavo Reyes-Terán

**Affiliations:** 1 Instituto Nacional de Enfermedades Respiratorias, Mexico City, Mexico; 2 Instituto de Biotecnología, UNAM, Cuernavaca, Morelos, Mexico; 3 Instituto Nacional de Diagnóstico y Referencia Epidemiológica, Mexico City, Mexico; 4 National Microbiology Laboratory, Public Health Agency of Canada, Ottawa, Canada; 5 Department of Medical Microbiology, University of Manitoba, Manitoba, Canada; 6 National Centre for Foreign Animal Disease, Canadian Food Inspection Agency, Ottawa, Canada; 7 Centro de Investigaciones en Enfermedades Infecciosas, Instituto Nacional de Enfermedades Respiratorias, Tlapan 4502, CP 14080, Mexico City, Mexico

## Abstract

**Background:**

Pandemic type A (H1N1) influenza arose in early 2009, probably in Mexico and the United States, and reappeared in North America in September for seven more months. An amino acid substitution in the hemagglutinin (HA), D222G, has been reported in a significant proportion of patients with a severe and fatal outcome. We studied the prevalence of HA222 substitutions in patients in Mexico during the second wave and its association with clinical outcome and pathogenicity in a mouse model.

**Methods:**

The nucleotide sequences of hemagglutinin (HA) from viruses collected from 77 patients were determined including 50 severe and fatal cases and 27 ambulatory cases. Deep sequencing was done on 5 samples from severe or fatal cases in order to determine the quasispecies proportion. Weight loss and mortality due to infection with cultured influenza viruses were analyzed in a mouse model.

**Results:**

Viruses from 14 out of 50 hospitalized patients (28%) had a non aspartic acid residue at the HA 222 position (nD222), while all 27 ambulatory patients had D222 (p = 0.0014). G222 was detected as sole species or in coexistence with N222 and D222 in 12 patients with severe disease including 8 who died. N222 in coexistence with D222 was detected in 1 patient who died and co-occurrence of A222 and V222, together with D222, was detected in another patient who died. The patients with a nD222 residue had higher mortality (71.4%), compared to the group with only D222 (22.2%, p = 0.0008). Four of the 14 viruses from hospitalized patients were cultured and intranasally infected into mice. Two viruses with G222 were lethal while a third virus, with G222, caused only mild illness in mice similar to the fourth virus that contained D222.

**Conclusions:**

We confirm the elevated incidence of HA222 (H1N1)pdm09 variants in severe disease and mortality. Both clinical and mouse infection data support the idea that nD222 mutations contribute to increased severity of disease but additional determinants in disease outcome may be present.

## Introduction

The first pandemic of this century originated as a novel influenza A/H1N1 virus (pdm09) in 2009. This virus was first detected in March and April in California and several cities of Mexico [1,2]. A second wave included those cases occurring from late August 2009 and March 2010 [3]. As of August 2010, more than 214 countries had reported laboratory confirmed cases of pdm09, including over 18,449 deaths [4]. Although most confirmed cases resulted in uncomplicated influenza illness, some patients required hospitalization due to severe pneumonia and respiratory failure, with a fatal outcome in some cases [5]. The factors that control the generation of pathogenicity in this novel influenza virus remain unknown. However the ability of influenza viruses to activate innate immunity [6], induce apoptosis mediated by the PB1-F2 protein [7], and contributions of the viral RNA polymerase proteins [8], the NS1 and M2 proteins [9,10], and viral receptor binding preference [11] could be important. The tissue tropism and the binding of influenza viruses to their target cells is mediated by the viral HA protein which recognizes cell surface glycoconjugate receptors that terminate in sialic acid (SA) residues. It has been described that single amino acid changes can alter receptor specificities, and amino acid changes in HA D222G have been associated with acquisition of affinity for *N*-acetylneuraminic acid with an α-2,3 ketosidic linkage to galactose (SA α2,3Gal), increasing the infection of lower respiratory cells and likelihood of viral pathogenicity [12].

HA D222G substitution or different variants (D222N, D222S) has been observed in pdm09 viruses isolated from fatal and mild cases in several countries [13,14]. The first report of substitutions for D222 showed considerable frequency of change at this position (18%) only in fatal and severe cases in Norway [15]. In contrast, two later studies found D222G substitution in mild cases, with Hong Kong and Greece reporting 4% and 15%, respectively [16,17]. The effect of these substitutions on viral pathogenicity in animal models is also unclear. In the most recent report [18] a recombinant pdm09 with the D222G substitution showed increased virulence in mouse model but was not lethal in the mouse or ferret model.

In the present work we describe a high prevalence (28%) of the D222G substitution in coexistence with D222N and D222S in 50 pdm09 positive patients admitted through the emergency room at the National Institute of Respiratory Diseases in Mexico City during the second pandemic wave. Viruses containing mutations in HA position 222 were associated with higher mortality. In addition, two of three isolated viruses with mutations in this position were lethal in mice. Substitutions at D222 might be associated with increased pathogenicity in humans and also in mice, but the current results suggest that additional unknown determinants also contribute to pathogenicity.

## Results

### Detection of variants in HA 222 position in severe and fatal cases

The sequences obtained by the Sanger method from three or four different amplifications of each sample of the partial HA gene indicated that 14 out of 50 hospitalized patients (28%) had a non aspartic acid residue at the HA 222 position (GenBank number accession CY100425-CY100438), while none of 27 outpatients had a substitution at this position. The rate of non aspartic acid HA 222 residues for hospitalized vs. ambulatory patients was significantly higher (p = 0.0014). Four samples had a RRT codon (patients INER014, INER047, INER068 and INER405), assuming R is the ambiguity code for A and G, meaning that these viruses could have AAT (N), AGT (S), GGT (G) or GAT (D) at this position. Next generation sequencing was done for samples INER047 and INER405 and the quasispecies proportion was determined (see data below). Five samples had wild-type D222 coexisting with G222 (INER088, INER111, INER264, INER307 and INER426), 3 had only a G222 substitution (INER114, INER147, INER306), 1 sample had wild type D222 coexisting with N222 (INER399) and finally 1 sample had wild type D222 coexisting with V222 and A222 (INER057) (Table 1). Of the 24 fatal cases, 10 presented a mutation in HA amino acid position 222. G222 was detected as sole variant or as a covariant at position 222 in 8 patients. N222 was detected in 1 patient who died and the variants V222 and A222 were also detected in 1 patient.

**Table 1:** **Pandemic influenza A** (**H1N1**) **viruses characterized by Sanger sequencing for amino acid position 222 of the HA**

**HA position 222 codon**	**Clinical outcome**		
	**Mild (n = 27)**	**Severe (n = 26)**	**Fatal (n = 24)**
**222D (wt)**	**100% (27)**	**84.6% (22)**	**58.2% (14)**
222D, 222E, 222G, 222N	0% (0)	7.7% (2)	8.4% (2)
222G	0% (0)	0% (0)	12.5% (3)
222D, 222G	0% (0)	7.7% (2)	12.5% (3)
222D, 222N	0% (0)	0% (0)	4.2% (1)
222D, 222V, 222A	0% (0)	0% (0)	4.2% (1)
All 222 variants	0% (0)	15.4% (4)	41.8% (10)

Clinical outcome based on patient information, assigned into categories by a medical specialist according to WHO guidance criteria.

### Clinical characteristics of hospitalized patients

When considering respiratory illness during an outbreak as our study population, and separating hospitalized patients as cases of severe manifestations and ambulatory patients as mild cases, we found that the proportion of the D222G mutation was significantly higher in the former group (14 of 50, 28%) than in the ambulatory patients (0%, p = 0.001, Table 1). Within the hospitalized patients, the mortality of the patients, which were cases with the mutation, was significantly higher than the patients without (p = 0.0008).

Additionally there was a significantly higher use of mechanical ventilation in the patients with a non D222 residue (p < 0.0001) when comparing with hospitalized patients with D222 only (0.0197). Overall, 41.7% of patients that died and 37.5% of patients that required mechanical ventilation presented variants in residue 222, whereas only 7.5% of the survivors and 4.5% of the patients that did not require mechanical ventilation had these variants.

Clinical characteristics of the patients are shown in Table 2. There were no significant differences in demographics such as age, gender or body mass index. The clinical presentation was worse in patients with variants in the HA 222 position, exhibiting greater neutropenia and lymphopenia (Table 2). All hospitalized patients suffered from multifocal pneumonia. Among other co-morbidities analyzed, patients with non D222 variant viruses had higher systemic arterial hypertension when compared to patients with only D222 virus (p = 0.0278) (Table 2). The time from onset of symptoms to hospitalization were similar in both groups (Table 2).

**Table 2:** Clinical characteristics of hospitalized patients and co morbidities identified in hospitalized patients

**Variable**	**222D**	**222G/N/V/A**	**p**
Female gender	33.33%	66.67%	0.0884
Age (yrs)	40.5 (26–50.5)	39.5 (29.5 - 47.5)	0.9145
BMI	30.57 (26.93 - 33.06)	35.77 (27.69 - 38.02)	0.359
Evolution time (days)	7 (4–8)	4.5 (4–6.5)	0.2102
Heart rate (bpm)	110 (84.5 - 120)	108 (90.5 - 117.5)	0.8702
Resp Rate	28 (22–32)	24 (22–28)	0.1951
O2 saturation (skin)	83 (64.5 - 87)	70 (60–78.5)	0.0793
O2 saturation (blood)	84 (68.9 - 88.6)	73.35 (67.6 - 82.6)	0.168
fiO2	21 (21–28)	21 (21–76.25)	0.1668
Kirby score	192.14 (116.71 - 235.72)	170.95 (73.5 - 216.43)	0.2
APACHE II	9 (7–12)	8 (6.25 - 11)	0.6787
SOFA score	4 (2–7)	4 (3.25 - 6)	0.6646
Alkaline phosphatase	105 (78.25 - 136.75)	94 (87.5 - 157.5)	0.5886
Platelets	183500 (128500–236500)	130500 (107000–176500)	0.1275
Albumin	3.4 (3–3.87)	2.9 (2.48 - 3.44)	0.0509
Leukocyte count	6800 (4650–9150)	4450 (3300–5050)	0.0079*
Neutrophyl count	5750 (3700–8460)	3550 (2700–4300)	0.0079*
lymphocyte count	795 (500–1050)	400 (300–500)	0.031*
Hemoglobin	15.25 (13.3 - 15.85)	12.65 (10.75 - 13.35)	0.0017*
**Co morbidities**	**222D**	**222G**/**N**/**V**/**A**	**p**
Obesity	13.89%	8.33%	0.9999
Diabetes mellitus	40.5 (26–50.5)	39.5 (29.5 - 47.5)	0.9145
Systemic arterial hypertension	5.56%	33.33%	0.0278*
Asthma	11.11%	0%	0.5597
Pseudomonas Sp.	8.33%	0%	0.5629
Staphylococcus Sp	11.11%	8.33%	0.9999
Other	*Acinetobacter baumannii*, *Klepsiella pneumonie*, HIV (2), COPD, pulmonary fibrosis, unspecified mycobacteria	Down syndrome	

Values are medians (interquartile range).

* statistically significant.

**Figure 1:**
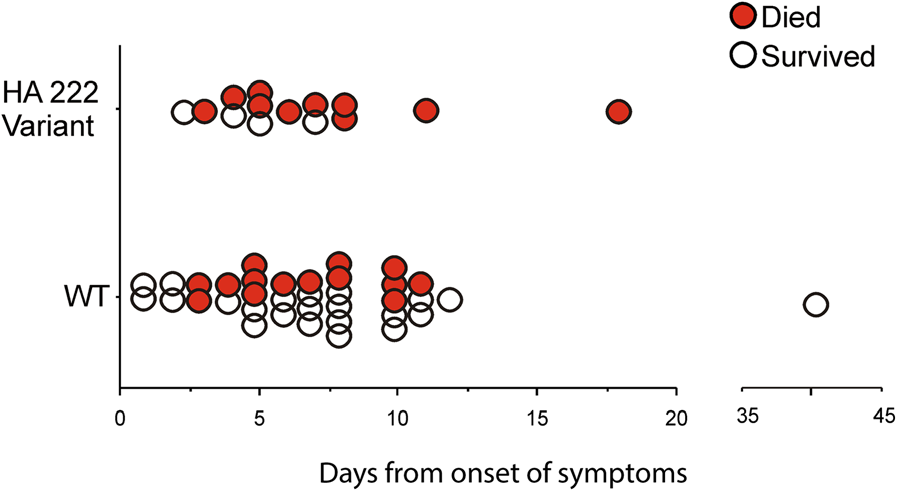
**Time from onset of symptoms to sample time at the hospital**, **for patients with and without the D222 variants.** WT: wild type (222D). There were no statistical differences (p = 0.4055).

To determine if D222 variants emerged in patients with longer evolution time, we analyzed the days from onset of symptoms to sample time at the hospital admittance and the presence or absence of D222 variants. There was no statistical difference (p = 0.4055) between patients with variants in the 222 position and patients without these substitutions (Figure 1).

### Genetic variations at positions 715–716 of HA nucleotide sequences

**Figure 2:**
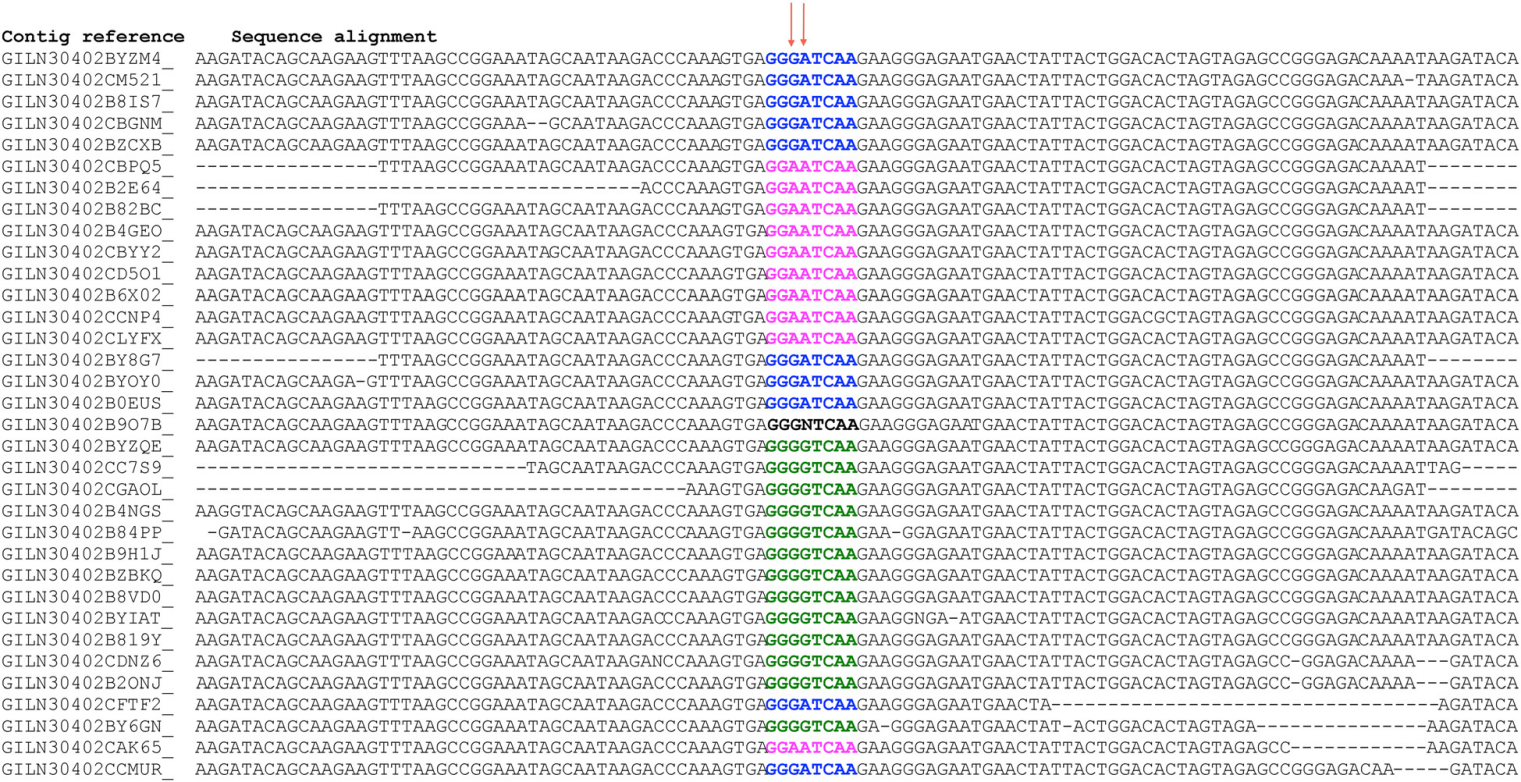
**D222 variants obtained by deep sequencing of sample 405.** Schematic representation of two nucleotide variations, 715 and 716 (N1 position), arrows indicate both positions of the HA sequence, and the alignment image of the 100-mer reads. Every read shown in blue wild type GAT (D), in pink AAT (N), in green GGT (G) and in black undefined codon. Reference sequence was A/Mexico/InDRE4487/2009(H1N1).

To determine the genetic variations at nucleotide positions 715–716 of the HA open reading frame (ORF) coding for amino acid 222 and to analyze the complete sequence of some influenza viruses with these variants, two different strategies of next generation sequencing were used. We performed sequencing using HA amplicons of sample INER405. The GS Sequencer FLX Titanium platform produced 33,807 reads from a cDNA library generated from the clinical specimen, of which 3,870 were viral sequences after the reads were filtered for human sequences. To identify heterogeneous populations, the sequencing reads of HA were mapped against an influenza reference sequence using GS Reference Mapper (Figure 2). At codon 222 we found 29% of GAT, 38.2% of GGT, 29% AAT and one read had an undefined codon.

The whole genome of four influenza viruses was also determined by Illumina Genome Analyzer IIx for viruses isolated from patients with a severe and fatal outcome, and whose variants at nucleotide position 715–716 had previously been determined by Sanger sequencing (INER047, INER264, INER306 and INER307, GeneBank number accession CY100461-CY10068, CY100519-CY100542). With this strategy, 3.8 ×10^6^ to 8 ×10^6^ total reads per sample were obtained, of which 1.7 ×10^6^ to 4.6 ×10^6^ reads corresponded to the influenza reference genome. The codon at position 222 of HA of INER047 had 5% GAT (D), 32.5% GGT (G) and 62.5% AAT (N). Sample INER306 had 93% GAT (D) and only a few reads of GGT (G) and AAT (N) in the codon for residue 222. These findings were in agreement with the Sanger sequencing in samples INER047 and INER405; however three samples (INER264, INER306 and INER307) were found to contain >93% of the triplet GAT (D) by metagenomic analysis.

### Detection of other polymorphisms in influenza viruses of severe and fatal cases

In order to detect other mutations that could determine pathogenicity of these isolates we analyzed the entire genome of 5 samples (INER047, INER264, INER306, INER307 and INER405) that were isolated from patients with severe or fatal outcome. Several non-synonymous substitutions as compared to A/California/04/2009 (H1N1) (accession number GQ280797.1) were detected in every segment (Table 3). Seventeen substitutions were observed in three or more samples. Some of these substitutions (G172R and A203T in HA, L95P and V180I in NS1, Q7R and L106F in NS2, V106I and T381N in NA, P224S in PA and Q194H in PB1) were reported in GenBank for only a small number of pdm09 from Mexico and the United States. G172R presented polymorphisms in samples INER047, INER264 and INER306. HA codon 172 of sample INER047 had 78% of GGA (G) and 22% AGA (R), INER264 had 43% GGA (G) and 57% AGA (R), while INER306 showed 75% GGA (G) and 25% AGA (R). None of the viruses described in the present study had the amino acid change from histidine to tyrosine at residue 274 of the NA, associated with resistance to oseltamivir.

**Table 3:** **Non**-synonymous substitutions* **detected in 5 samples with 222 variants** (**INER047**, **INER264**, **INER306**, **INER307 and INER405**)

**Gene**	**Substitution**	**Subject:**				
		**INER047**	**INER306**	**INER405**	**INER264**	**INER307**
PB1	V587G	X	X	X		X
	A652V	X	X	X	X	X
PB2	E191G		X			
	Q194H	X	X	X	X	X
PA	P224S	X	X	X	X	X
	A307G	X				
	S395R		X			
	P434L		X			
	V521I			X		
	I573Y			X		
NP	S69P				X	X
	V100I	X	X	X	X	X
	T373I				X	
NA	V106I	X	X	X	X	X
	N248D	X		X		
	V304A					X
	T381N		X		X	X
M1	I24V		X			
	S30R				X	
	H222N	X				
NS2	Q20R		X	X	X	X
	L58F	X				
	L120F		X	X	X	X
NS1	V65M			X		
	L95P		X	X	X	X
	I123V	X	X	X	X	X
	I128T	X				
	V180I		X	X		X
	L181F				X	
	P215L	X				
HA**	N27D		X			
	N48D		X	X		X
	V169I		X			
	G172R	X	X		X	
	S179N		X	X		X
	W194R				X	
	A203T		X	X	X	X
	T214A	X	X	X	X	X
	I338V	X	X	X	X	X
	Y528C				X	

* Sequences used for comparison were from Influenza A virus (A/California/04/2009(H1N1)) accession number GQ280797.1.

** HA positions are given without removing the signal peptide.

### Pathogenesis of HA 222 variants in mice

All 14 clinical specimens from hospitalized patients were placed in MDCK cell culture in an attempt to derive a stock of virus from each. Four of the viruses (INER047, INER111, INER307 and INER405) were successfully cultured and a stock of virus was generated and titered on MDCK cells. Analysis of the viral variants in each stock from 20 different plaques of each showed that two of four isolates (INER111 and INER405) had only glycine at position 222, while INER047 showed nineteen viruses with glycine and one virus with asparagine. All viruses of sample INER307 had D222. The HA sequencing data suggests that culturing of the clinical isolates on MDCK cells tended to preferentially select one of the variants from each that had been identified in the analysis of HA quasispecies variability in the original clinical isolates. Complete sequencing of all other genes from the INER307, INER405 and INER047 MDCK-grown virus stock (GeneBank number accession KC261306-KC261337) indicated no changes associated with adaptation to cell culture compared to the sequences obtained from the clinical isolates (data not shown). The complete sequence of the MDCK-grown virus INER111 was also determined but the sequence of the clinical isolate was not available for comparison.

**Figure 3:**
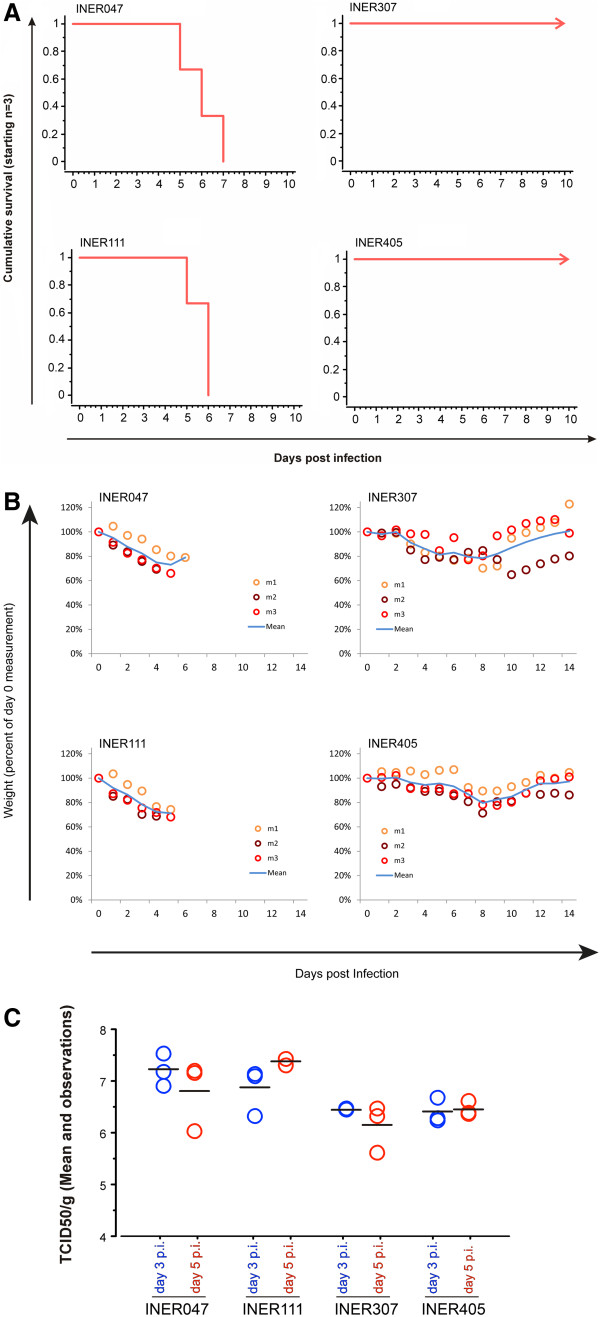
**Pathogenicity of pdm09 isolates in mice. A**) Cumulative survival curves in mice after intranasal inoculation with different viruses isolated from hospitalized patients are shown. Three mice per group were inoculated with different viruses: INER307 (D222), INER047 (G222:N222, 19:1), INER111 (G222) and INER405 (G222). Mice were monitored for 14 days. **B**) Mean body weight and observations in mice infected intranasally with 5.5 TCID _50_ of viruses isolated from hospitalized patients. Body weight losses were determined daily up to day 14 after inoculation. **C**) Viral titer were determined by TCID_50_ in lung homogenates of mice infected by viruses from the hospitalized patients.

As all four of the virus stocks had been derived from patients that had required hospitalization due to the severity of their illness, it was of interest to analyze the pathogenicity of infection with each virus in a mammalian animal model of infection. Intranasal infection of mice with each revealed differential pathogenesis; INER047 and INER111 viruses were lethal with rapid weight loss. Mice infected with viruses INER047 died at 4–6 days post-infection (dpi), whereas mice infected with virus INER111 died at 4 and 5 dpi (Figure 3A). INER047 was isolated from a patient that died and INER111 was from a patient requiring mechanical ventilation and extended hospitalization. Mice infected with all of the viruses lost ≥ 18% body weight, however mice infected with viruses INER307 and INER405 recovered the body weight by 13 dpi (Figure 3B). The 50% mouse lethal dose (MLD_50_) for viruses INER047 and INER111 were 4.0 and 3.0 TCID_50_ (expressed as the log_10_ of the 50% tissue culture infectious dose) respectively, whereas INER307 and INER405 were not lethal even at the highest doses used for infection (maximal dose of 6.5 TCID_50_). All of the viruses replicated efficiently in mouse lungs and high titers persisted until at least 5 dpi. The titers associated with viruses INER047 and INER111 causing lethal infection tended to be higher on both days than titers induced by viruses INER307 and INER405 that were not lethal (Figure 3C).

### Mouse pathology

**Figure 4:**
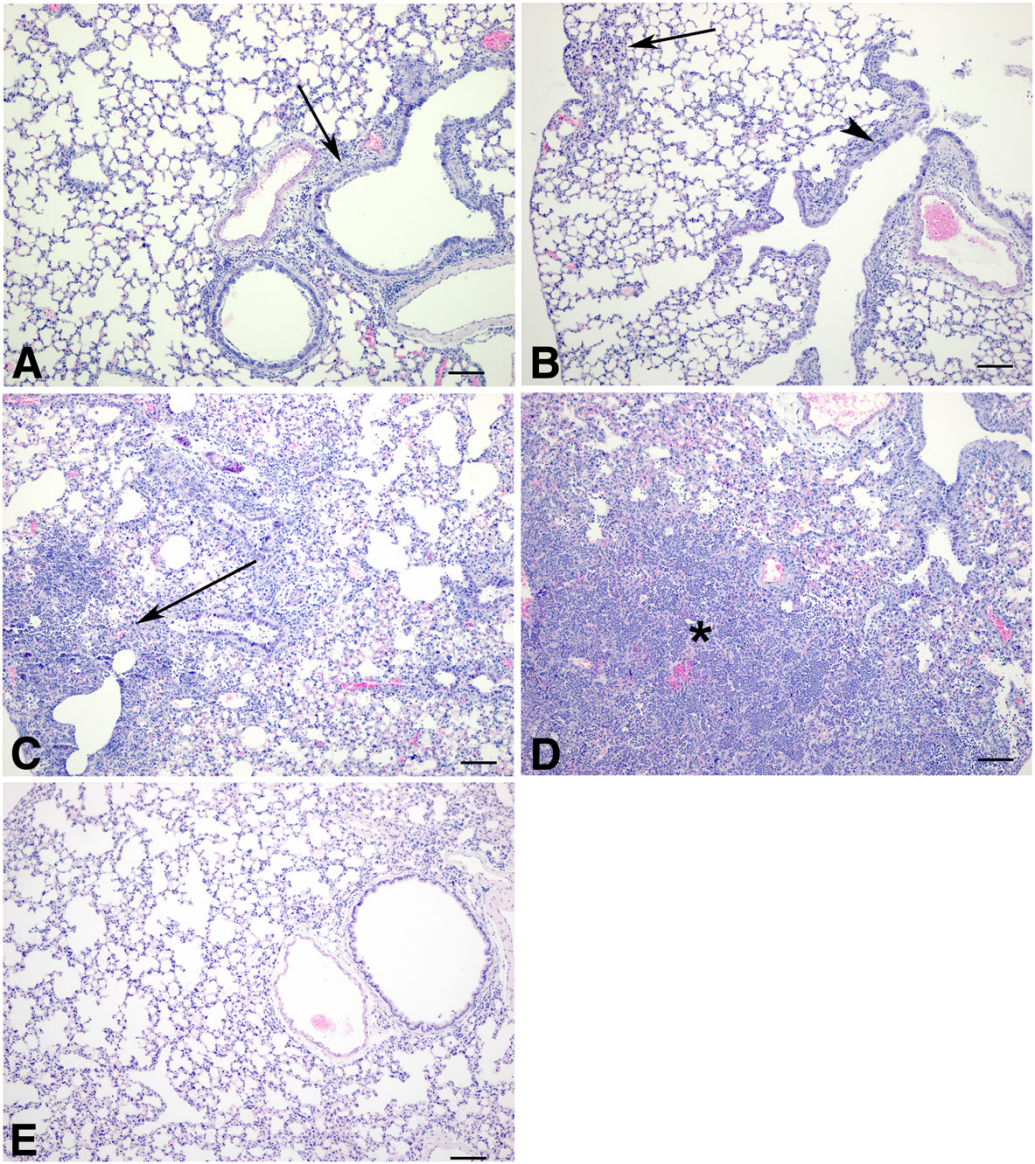
**Lung pathology of mice infected with various strains of H1N1 at 5 dpi. A**. Lung from an animal infected with strain INER307 showing that lesions are limited to bronchioles with necrosis, epithelial hyperplasia and peribronchiolar infiltrates of lymphocytes and plasma cells (arrow). H&E stain. **B**. Lung from an animal infected with strain INER405 shows bronchiolitis (arrowhead) and small areas of alveolar involvement (arrow). H&E stain. **C**. Lung from an animal infected with strain INER111 showing widespread inflammation and necrosis of alveolar walls. Note small area of consolidation with neutrophilic infiltrate (arrow). H&E stain. **D**. Lung from an animal infected with strain INER047 showing large areas of consolidation due to neutrophilic infiltrate (*). H&E stain. **E**. Lung from a non-infected control animal. H&E stain. Bar = 100 μm.

All of the viruses replicated efficiently in mouse lungs and high titers persisted at least until day 5 PI. The titers associated with viruses INER047 and INER111 causing lethal infection tended to be higher on both days than titers induced by viruses INER307 and INER405 that were not lethal (Figure 3C). Lesions in lungs of animals infected with strains INER307 and INER405 were similar and were primarily limited to the bronchioles. However, in addition to the presence of bronchiolar lesions, lungs from animals infected with strains INER111 and INER047 showed a diffuse pneumonitis involving most of the examined lung tissue. At 3 dpi, lesions were most often limited to the bronchioles with only scattered involvement of the alveolar walls (data not shown). Although there was still some ongoing necrosis of bronchioles at 5 dpi, many bronchioles showed epithelial hyperplasia typical of a regenerative process (Figure 4A and Figure 4B). Lesions observed in animals infected with strain INER111, at both 3 and 5 dpi, showed a more severe necrotizing bronchiolitis with many bronchioles showing complete loss of epithelium. In addition, there was a diffuse pneumonitis characterized by expansion of alveolar walls by inflammatory cells and edema with multifocal areas of necrosis (Figure 4C). At 5 dpi in lungs of animals infected with strain INER047, in addition to the pneumonitis observed at 3 dpi (data not shown) approximately 50% of the section was consolidated and lung tissue was almost completely replaced by neutrophils (Figure 4D). Lesions were not observed in non-infected control animals (Figure 4E).

**Figure 5:**
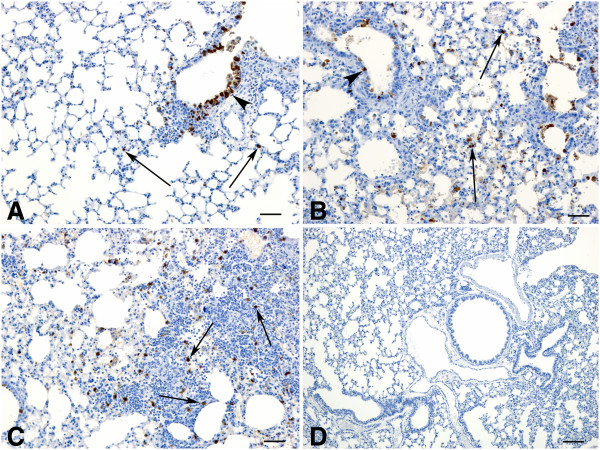
**Immunohistochemical detection of influenza A specific antigen** (**NP**) **in mice infected with various strains of H1N1 at 5 dpi. A**. Lung from an animal infected with strain INER405 (also representative of INER307 infection) showing detection of viral antigen in bronchiolar epithelial cells (arrowhead) and occasionally in cells within alveolar walls (arrows). Bar = 50μm **B**. Lung from an animal infected with strain INER111 showing viral antigen within bronchiolar epithelium (arrowhead) as well as extensively within alveolar walls (arrows). Bar = 50μm **C**. Viral antigen could be detected extensively throughout areas of consolidation (arrows) in an animal infected with strain INER047. Bar = 50 μm. **D**. Immunohistochemistry for influenza A specific antigen (NP) on lung from a non-infected control animal. Bar = 100μm.

As indicated by immunohistochemical detection of viral antigen (nucleoprotein; NP), an extensive distribution was found in bronchioles and only occasionally in macrophages and pneumocytes within alveolar walls of lungs from animals infected with strain INER405 (Figure 5A) similar to that observed for INER307 (data not shown). For strains INER047 and INER111 viral antigen was detected in bronchiolar epithelium as well as extensively within alveolar macrophages, necrotic debris within alveolar spaces and within pneumocytes (Figure 5B). Within areas of consolidation, antigen could be detected in numerous cells with the morphological appearance of macrophages or pneumocytes (Figure 5C). Viral antigen (NP) was not observed in non-infected control animals (Figure 5D).

## Discussion

D222G is the main substitution found in reports associated with severe or fatal cases of pdm09 [15,19]. Here we found this substitution only in viruses isolated from severe cases; we also found a correlation between the risk of death and HA 222 variants. Low white blood cell (WBC) levels and low hemoglobin levels correlated with the presence of HA 222 variants, and suggested that the viruses harboring variants in HA 222 could be associated with a more severe respiratory disease. Not all severe or fatal cases had this mutation but its emergence could intensify the severity of the disease.

A recent study found a delayed clearance of virus in patients with severe disease [20], which may represent an opportunity for the accumulation of different variants (including D222G) in patients. However in this study we found no correlation between duration of disease and emergence of D222 variants, supporting the argument that the substitutions appeared spontaneously and sporadically.

Respiratory tract cells expressing α2,3 receptors are more abundant deeper in the human airway, an observation that has led to the hypothesis that viruses with dual receptor specificity can replicate to higher titers in lungs, resulting in a more severe course of disease [11]. Recent studies suggest an increased receptor affinity of the 222G variant for ciliated bronchial epithelial cells, which may have an impact on disease severity [21]. Some studies indicate that such variants were also found more frequently in specimens obtained from the lower respiratory tract than from the nasopharynx [19]. Our findings indicate that the 222 variants including G222, N222, V222 or A222 were present in nasopharynx, showing their presence in the upper respiratory tract. Consistent with the presence of these variants in the upper respiratory tract, transmission of influenza virus with D222G from a patient with severe disease to a family member has been reported [22]. Other studies found that virus with D222G had acquired the ability to bind to SA α2,3Gal, retaining affinity for SA α2,6Gal, the preferential receptor of human viruses that is associated with attachment to cells of the upper respiratory tract [23]. Viruses with G222 do have dual tropism, perhaps resulting in more efficient infection of the lower respiratory tract, causing more severe disease. D222G, D222N, and D222E changes have been found in viruses isolated in different countries [15,19,22,24], but the D222V amino acid change has not been previously reported. A sequence of HA with A222 has been submitted to GenBank, but without any published clinical data. Changes in HA amino acid position 222 located in the receptor-binding cavity might potentially influence receptor binding of the influenza virus. Changing an acidic and polar amino acid (D) to neutral and non-polar (G, V and A) residue could affect the tropism of these new variants. Detection of HA 222 variants has been realized using both pyrosequencing and Sanger methods, resulting in different proportion of polymorphisms in that position [25], in our case we found discrepancies also between both methodologies, due perhaps an enrichment of minority population during PCR used to amplify metagenomic sequencing library, especially given low amount of RNA used.

We also found that HA substitution G172R, which co-segregated with HA amino acid 222 variants, was present in fatal cases only. This substitution is located in the HA antigenic site Sa that could be implicated in antigenic changes of HA [26]. In fact, future amino acid substitutions in the antigenic sites of pdm09 HA (Gly_172_Glu) have been anticipated [26]. Furthermore, a HA variant with G172E was found in one fatal case [27]. The biological significance of the presence of both substitutions and their relationship with severity remains unknown.

The severity of the disease observed in human patients was generally consistent with the disease observed in mice. The virus INER047 isolated from a patient with a fatal outcome was lethal at 4 to 6 days PI in mice; and the virus INER111 that caused severe disease in a patient requiring mechanical ventilation was also lethal in mice at 4 and 5 days PI. However, the virus INER405 that had 222G in HA was of limited pathogenicity in mice. This virus was isolated from a patient who recovered after being hospitalized for one month showing an association between the virus and severe infection. However, the mouse study results suggest that additional determinants of pathogenicity in mammals likely exist, aside from 222G in HA, that might be responsible for severe disease in humans but are not determinants of disease in mice. This data suggests that there might be pathogenic factors affecting the clinical course of the disease in humans that have not been described. A recent report in an animal model showed that a pdm09 D222G virus caused more severe disease with fatal outcome in mice, compared with the wild type influenza virus [23,28], although experiments with ferrets did not support a causal link of D222G substitution with virulence [18].

Here we report a high prevalence of pdm09 HA codon 222 variants in severe and fatal cases of pneumonia in Mexico City during the second wave of the 2009 influenza pandemic. Recent data confirmed an increase in the overall mortality in Mexico during the pandemic and also mortality due to respiratory and cardiovascular causes, especially in young and middle age individuals [29]. Our results support the suggestion that substitution at HA position 222 is associated with pdm09 pathogenicity, with variants appearing spontaneously during the two pandemic waves in Mexico. Some reports and sequence data have demonstrated that 222 variants appeared in the early phases of the pandemic, increased during 2009–2010 influenza season and have reduced to their present levels during 2010–2011 and 2011–2012 influenza season [30,31]. Bacterial infection or coexisting conditions do not appear to be major contributing factors to the severity (Table 2). We have identified additional viruses with 222 variants (D222V and D222A) in the same individual, but the biological importance of these substitutions in tropism and pathogenicity remains to be elucidated. The disease observed in animal models was generally concordant with the clinical outcome of patients, but additional determinants of virulence may contribute to the increased severity of disease observed in mammals and is a significant point for further investigation of viral determinants of severe disease in humans caused by pdm09 viruses.

## Material and methods

### Patients

Respiratory samples were obtained from all patients who were hospitalized at the National Institute of Respiratory Diseases in Mexico City, during September 2009 to April 2010. Twenty-seven patients that sought medical care at our institution during the same period of time, but that did not require hospitalization were also included. Nasopharyngeal swabs were collected and infection by pdm09 was assessed by a real-timereverse-transcriptase–polymerase-chain-reaction (RT-PCR). This study was approved by the Bioethics and Research Committee of the National Institute of Respiratory Diseases in Mexico (B-0411). Written informed consent was provided by study participants and/or their legal guardians.

### Sanger sequencing of HA

Viral RNA was extracted from 200 μL of respiratory samples using a high throughput system (MagNA Pure®, Roche, Indianapolis, IN, USA). RT-PCR was performed with the one-step RT-PCR kit (QIAGEN, Valencia, CA, USA). The HA gene was partially sequenced; briefly, the initial amplifications were performed using specific primers for pdm09: HA 58F 5^′^ TTATGTATAGGTTATCATGCGAA 3^′^ and HA 1676R 5^′^ ACCCATTAGAGCACATCCAGAAAC 3^′^, a second round PCR reaction were performed with HA 149F 5^′^ TAGAAGACAAGCATAACGGGAAA 3^′^ and HA 865R 5^′^ CTGGTGTATCTGAAATGATAATA 3^′^ (608 bp). Nucleotide sequences of PCR products were determined using BigDye® Terminator cycle sequencing kit (Applied Biosystems, Foster City, CA, USA) and were analyzed with the ABI Prism 3100 analyzer sequencer (Applied Biosystems). Nucleotide sequences of PCR products were aligned and analyzed using Clustal X and MEGA 4.0 respectively.

### Illumina next generation sequencing and sequence analysis

Whole genome of influenza A isolates was amplified in an one tube multisegment RT-PCR reaction as previously reported [32]. Products obtained were purified and concentrated and used as input material in constructing multiplex libraries using Illuminas Genomic DNA sample Prep Kit with multiplexing primers. Three hundred base-pair sized libraries were loaded in a flow cell (four libraries per lane) from a Genome Analyzer IIx (Illumina, San Diego, CA). Sequencing was performed through 46 cycles of single base pair extensions, followed by acquisition of multiplex code. Image analysis was done using goat.py, and the base calling was done using Bustard.pl, both programs included in Off-Line Basecaller Software v1.6 (Illumina, Hayward, CA). Processing of sequence data was carried out using Maq version 0.7.1 [27] and R/Bioconductor [33] programs. All reads were quality filtered using the Off-Line basecaller Software v1.6 (Illumina, San Diego, CA). Reads were aligned to reference influenza A genome A/Netherlands/602/2009, with maximum of 5 mismatches allowed.

### GS Sequencer FLX Titanium platform

For sample INER405, the HA gene was amplified using a pair of specific primers: MBTuni12, HAREV1166 (5^′^ GGCATTCTGTGTGCTCTTCA 3^′^) and MBTuni13, HAFW756 (5’ AGCCGGGAGACAAAATAACA 3’). A single stranded DNA library was prepared with the GS Titanium General Library Preparation kit (Roche) as specified by the manufacturer. The DNA library was clonally amplified by emulsion PCR (emPCR) with the GS Titanium emPCR kit (Roche). The amplified beads were recovered and enriched and approximately 340,000 beads were loaded on one line and sequenced in the PicoTiterPlate using a GS Titanium Sequencing reagents kit XLR70. All sequencing reads, were assembled separately in GS De novo Assembler Version 2.0.00. The resulting contigs were analyzed using NCBI megablast program, mapping against an influenza reference sequence (A/Mexico/InDRE4487/2009(H1N1)).

### Mouse study

Influenza viruses were isolated from respiratory samples in MDCK cells (ATCC # CCL-34). Titers of each stock virus were determined as the 50% tissue culture infectious dose (TCID_50_) and reported as the log_10_ of the TCID_50_ value. Each of the viruses was plaqued on MDCK cells and 20 plaques of each were picked and stocks were produced in MDCK cells. Viral RNA was isolated from each plaque virus stock and the HA sequenced in the region of amino acid 222. To determine the 50% mouse lethal dose (MLD_50_), serial 10-fold dilutions of each virus, starting at 6.5 TCID_50_, were intranasally (i.n.) inoculated in 3 anesthetized (isoflurane) mice/dilution. The MLD_50_ was expressed as the log_10_ of the TCID_50_. Calculations for TCID_50_ and MLD_50_ were done according to the method of Reed and Muench [34].

Groups of twelve 5-6-week-old female BALB/c mice (Charles River) were infected i.n. with 5.5 TCID_50_ of each virus. Animals were weighed daily for 14 days and monitored for clinical signs. Three mice per group were sacrificed on days 3 and 6 post-infection and lungs were removed aseptically. For determination of viral titers, lung tissues were harvested, weighed, homogenized and supernatants were titrated in 10-fold serial dilutions on MDCK cells. Titers were expressed as log_10_(TCID_50_) per gram of tissue.

### Pathology and immunohistochemistry

Lung samples were fixed in 10% neutral buffered formalin, embedded in paraffin, sectioned at 5 μm and stained with haematoxylin and eosin for examination by light microscopy. For immunohistochemistry, paraffin tissue sections were quenched for 10 minutes in aqueous 3% H2O2 then pretreated with proteinase K for 15 minutes. Slides were blocked for 30 minutes with Rodent Block M from MM horse radish peroxidase (HRP)-polymer kit (Biocare Medical, USA) then rinsed with TBST. The primary antibody was a mouse monoclonal antibody specific for influenza A nucleoprotein (NP) (F26NP9, produced in-house) and was used at a 1:10,000 dilution for one hour. They were then incubated for 15 minutes with MM polymer-HRP (Biocare Medical, USA) and reacted with the chromogen diaminobenzidine (DAB). The sections were then counter stained with Gill’s hematoxylin.

All animal experiments were performed with strict adherence to the recommendations of the Canadian Council on Animal Care (CCAC). The animal use document was approved by the Animal Care Committee (AAC) of the National Microbiology Laboratory, Canada.

### Statistical analysis

Effects were evaluated by Wilcoxon’s Rank Sum test, odds ratio (ORs) estimated by normal approximation for small samples, and significance assessed by Fisher´s exact method. Correlations were calculated with Spearman’s Rho. Data analysis was conducted using R statistical software, version 2.10.1. All tests were two sided and not controlled for multiple comparisons.

## Competing interests

The authors declare that they have no competing interests.

## Authors’ contributions

JAVP, PI, DK and JERG contributed to the study design. JAVP performed the RT-PCR real time assays, Sanger sequencing, analysis and interpretation of the data and wrote the manuscript. PI, MEZ and AGCG performed Illumina next generation sequencing. JERG, EGD and JOA performed GS Titanium next generation sequencing. YL and NB carried out isolation and characterization of influenza viruses. DK and CR carried out animal experiments and molecular studies. CE carried out pathology and immunohistochemistry studies. CEO and ESR carried out the statistical analysis and wrote the manuscript. DPRR, GBB, YAT, IL and CAA provided patients' samples, controls and summary of clinical data. GRT and RPP assisted with manuscript preparation, and helped to edit the manuscript. All authors read and approved the manuscript.
